# HCV Animal Models: A Journey of More than 30 Years

**DOI:** 10.3390/v1020222

**Published:** 2009-09-02

**Authors:** Philip Meuleman, Geert Leroux-Roels

**Affiliations:** Center for Vaccinology, Ghent University and Hospital, Building A, 1st floor, De Pintelaan 185, B-9000 Gent, Belgium; E-Mail: geert.lerouxroels@ugent.be

**Keywords:** HCV, animal model, chimpanzee, chimeric mice, uPA-SCID, hepatitis C, antiviral therapy, neutralizing antibodies

## Abstract

In the 1970s and 1980s it became increasingly clear that blood transfusions could induce a form of chronic hepatitis that could not be ascribed to any of the viruses known to cause liver inflammation. In 1989, the hepatitis C virus (HCV) was discovered and found to be the major causative agent of these infections. Because of its narrow tropism, the *in vivo* study of this virus was, especially in the early days, limited to the chimpanzee. In the past decade, several alternative animal models have been created. In this review we review these novel animal models and their contribution to our current understanding of the biology of HCV.

## 1. Introduction

In the mid-1970s, a rapidly increasing number of patients was diagnosed with a transfusion-associated form of hepatitis that could not be ascribed to an infection with hepatitis A virus, hepatitis B virus, Epstein-Barr virus, cytomegalovirus or any other virus known to cause liver inflammation [1,2]. Therefore these cases were termed non-A, non-B hepatitis (NANBH). Although many efforts were undertaken, it took nearly 15 years before the causative agent of NANBH was identified as the hepatitis C virus (HCV) [3]. Since then, our knowledge on this virus progressed relatively slowly, mainly due to the absence of suitable cell culture and animal models. Although a subgenomic replicon system was available to study the replication of the HCV genome [4], only very recently a complete cell culture system was developed in which all aspects of the viral life cycle could be studied [5–7]. Unfortunately this system is restricted to one specific viral isolate, termed JFH-1, or chimeric derivatives thereof [5,8]. In addition, this viral strain only replicates robustly in Huh7 or Huh7.5 hepatoma cells, which have characteristics that differ from these of primary human hepatocytes, the natural host cells of HCV [9].

Although cell culture models are very useful to study different aspects of HCV, one must always be cautious that the artificial cell culture conditions may have an influence on the results generated. To mimic more closely the natural situation, *in vivo* studies are needed. However, due to the narrow tropism of HCV, *in vivo* studies were for a long time restricted to the chimpanzee. Meanwhile several other species have been evaluated for their permissiveness for HCV infection, but most of them seemed resistant to infection. The development of some more complex animal models that were surgically transplanted with human hepatocytes were more successful and have already contributed considerably to our understanding of this virus. In this review we give an overview of the different animal models that have been used in the past few decades for the study of HCV.

## 2. Primate models

### 2.1. The chimpanzee

The animal that contributed the most to our knowledge on HCV is undoubtedly the chimpanzee. In fact, the chimpanzee played a pivotal role in the discovery of HCV. The viral genome of HCV was cloned from a chimpanzee that was experimentally infected with NANBH [3]. Chimpanzees share more than 98% of their genome sequence with humans and are therefore routinely used for the study of human diseases. However, despite this high genomic homology there are some clear differences. Chimpanzees and humans do not share any HLA class I alleles and clear differences within the MHC class II region are observed. These and other differences make that the disease pattern and outcome in chimpanzees are not necessarily the same as in humans. The chronicity rate of HCV in chimpanzees is only 39% to some [10], but 60% according to others [11–13], while in humans it is estimated to range between 70 and 85% [14]. In addition, the liver disease is significantly milder in chimpanzees: fibrosis and cirrhosis have never been observed and the development of hepatocellular carcinoma has only been detected in one animal [15].

Nevertheless, the chimpanzee proved very valuable for the study of the molecular, immunological and clinical aspects of HCV infection. While it is nearly impossible to study the acute phase of HCV infection in humans, because of the usual absence of any specific symptoms early after infection, experimental HCV infections in chimpanzees allow close and well-timed monitoring of viral kinetics, host response, disease manifestation and outcome [11,16,17]. In addition, frequent collection of liver biopsies allows for a detailed investigation of the intrahepatic innate and adaptive immune responses [13,18–22]. The knowledge gained from immunological studies in chimpanzees and humans has lead to the development of different candidate vaccines. Again, the chimpanzee proved invaluable in the evaluation of these candidate prophylactic and therapeutic vaccines [23–25].

Because of ethical and financial reasons, most chimpanzee studies were usually limited to one or only a few animals and nowadays it has become extremely difficult to perform any studies at all. Therefore alternative animal models were sought. Different other primates, like cynomolgus monkeys, green monkeys, rhesus monkeys, Japanese monkeys and the doguera baboon have been inoculated with HCV, but none of these species were susceptible to infection [26].

### 2.2. Callithricidae

In the eighties, several reports described successful HCV infection in two tamarin species: *Saguinus mystax* and *S. labiatus* [27–30]. However, the infection rate in these New World primates was disappointingly low and the experimental results were rather inconsistent. Later another tamarin species, *Saguinus oedipus oedipus*, proved resistant to an experimental HCV infection [31]. Although tamarins cannot be infected with HCV, they are susceptible to hepatitis GB virus B (GBV-B), a flavivirus that is phylogenetically closely related to HCV [32]. GBV-B causes an acute infection in tamarins, characterized by increased ALT levels and transient high viremia (> 10^9^ genome equivalents/mL). Although two tamarins evolved to chronicity after inoculation with GBV-B [33,34], the viral infection is usually cleared spontaneously between 10 to 26 weeks. The duration of viremia can be prolonged to 46 weeks if the animals are undergoing an immunosuppressive therapy at the moment of GBV-B infection [35]. GBV-B can also infect common marmosets (*Callithrix jacchus*) [36]. Likewise, these animals experience high viremia (10^8^ to 10^9^ genome equivalents/mL) for a period of 40 to 60 days after which the infection is usually cleared within 10 to 20 weeks postinfection [36,37]. Nonetheless, this surrogate model system has already proven valuable for the study of protective immunity [38] and the evaluation of antivirals [36]. Using chimeric HCV/GBV-B viruses it is possible to study the function of HCV genomic sequences and proteins [39–42].

### 2.3 Tree shrews

Tree shrews, originally considered to be lower primates but later classified into a separate order Scandentia [43], are small non-rodent mammals that also have been investigated for their permissiveness for HCV infection. Xie *et al.* showed that *Tupaia belangeri chinensis* could be infected with HCV, but unfortunately only a small subset (20–35%) of inoculated animals exhibited clear signs of infection. Viremia could only be transiently or intermittently detected at relatively low viral titers [44]. More recently, another group showed higher infection rates when animals were injected with human plasma samples as well as with cell culture derived virus [45]. Several animals even became chronically infected. There is no clear explanation for the discrepancies between these two studies. If tree shrews could indeed be reproducibly infected with HCV they would be a suitable alternative for the chimpanzee. These animals have the size of a small rat, adapt easily to the environment in animal facilities and retain a high reproduction rate in captivity. This results in a lower cost and overcomes the problem of wild tupaias that are frequently already infected with viruses, such as the tree shrew herpes virus which may cause spontaneous hepatitis [46].

## 3. Rodent Models

### 3.1. Rats

Wu and colleagues created a novel rat model by tolerizing immunocompetent rats to Huh7 cells by injecting these hepatoma cells through the uterine wall into the peritoneal cavity of fetal rats on day 17 of gestation [47]. The day after birth, the rats were transplanted with Huh7 cells and one week later they were inoculated with HCV. Viral infection persisted for several weeks but serum levels of HCV RNA reached maximum levels of only 2×10^4^ copies/mL. It is clear that viral replication can be achieved in this rat model, but the low level of engraftment, the use of hepatoma cells and the low viremia are major weaknesses. The mismatch between human and rat MHC molecules definitely prevents the study of adaptive immune responses towards the infected hepatoma cells. Moreover, the presence of a functional rat immune system excludes the possibility to transplant selected human lymphoid cells, like HCV-specific T-cell clones.

### 3.2. Transgenic mice

Transgenic mice are produced by embryo microinjection of gene constructs. In principle, any gene can be inserted and placed under the control of any specific promoter. Several HCV-transgenic mice have been created that contain the genetic code for one or more HCV proteins, usually to study liver pathology.

Koike *et al.* generated a transgenic mouse containing both viral envelope proteins E1 and E2 [48]. Long-term follow-up of these mice did not show any evidence of hepatocellular damage. Several other HCV transgenic mice, containing one or both of the envelope proteins in combination with the core gene, also did not suffer from any liver disease [49,50]. In contrast, different groups described progressive hepatic steatosis and hepatocellular carcinoma in HCV transgenic mice containing the genetic code of either the viral core protein, the nonstructural proteins or even the entire HCV open reading frame [51–58]. These discrepancies may be explained by a relationship between inflammation-associated hepatocarcinogenisis and the host genetic background [59].

Using NS5A-transgenic mice, Majumder *et al.* showed that this viral protein can inhibit TNF-mediated hepatic apoptosis by interference with the TNF-signal transduction pathway [60]. NS5A physically associates with TRADD (TNF-Receptor 1 Associated Death Domain protein), which prevents the association between TRADD and FADD (Fas-Associated Death Domain protein) and thereby blocks TRADD-mediated NF-kB activation. Sälberg’s group demostrated that NS3/4A-transgenic mice do not spontaneously develop liver pathology, but hepatocytes expressing NS3/4A become resistant to TNFα-induced liver damage. Treatment with a p38 MAP-kinase inhibitor can revert the TNFα resistance [61].

A general drawback of HCV-transgenic mice is that the transgene usually integrates at a high copy number at a random site in the host’s genome. In contrast to a natural infection, the viral proteins typically are highly overexpressed in an uncontrolled manner. Certain qualities attributed to the viral proteins therefore might be related to this artificial overexpression and/or may ensue from possible interference with the regulation of genes located near the integration site. The development of new transgenic mice in which the expression of the viral proteins can be controlled may to some extent overcome these limitations [62].

### 3.3. Trimera mice

The first human-mouse chimera suitable for viral hepatitis studies was the Trimera mouse. This mouse was originally developed for the engraftment of human hematopoietic cells and tissues [63,64]. The name ‘Trimera’ refers to the three different genetic backgrounds of the tissues used to create this mouse model. After lethal total body irradiation, normal or BNX (beige/nude/X-linked immunodeficient) mice are rescued with severe combined immune deficiency (SCID) bone marrow and subsequently small human liver fragments are transplanted under the kidney capsule or ear pinna. Ilan and coworkers demonstrated that the Trimera mouse could be used for the evaluation of compounds with possible anti-HCV activity [65]. For this, human liver fragments were infected with HCV *ex vivo* and transplanted under the kidney capsule of recipient mice. Untreated mice experienced an active viral infection that lasted for about one month, with viral titers ranging between 1×10^4^ and 6×10^4^ copies/mL. Treatment of these mice with an inhibitor of the HCV internal ribosome entry site resulted in a dose dependent reduction of the viral load during therapy. The efficacy of treatment was rather low, but this was probably more related to the qualities of this compound than to the characteristics of the animal model used. More recently, the same group has used the Trimera mouse for the preclinical evaluation of two antibodies with possible neutralizing activity against HCV [66]. Mice with proven infection that were treated with the antibodies experienced a 0,6 to 0,8 log_10_-fold reduction in viremia after five days. Both antibodies were also tested for their ability to prevent an HCV infection. Therefore, the viral inoculum was pre-incubated for two hours with both antibodies before it was exposed to the human liver sample. Several weeks after transplantation, circulating HCV RNA levels were approximately 1 log_10_-fold lower in treated mice compared to control animals. A phase I clinical trial in chronic HCV patients failed to show any significant change in viral load after treatment (www.xtlbio.com).

Although the Trimera mouse model may to some extent be useful for the evaluation of STAT-C (specifically targeted antiviral therapy for HCV) compounds, it has some severe shortcomings. The viral titers observed are usually very low and major histological changes within the transplanted tissue, like ischemia, fibrosis, loss of lobular architecture and necrosis, are routinely observed [65,67,68]. The occurrence of these histologic abnormalities is not surprising since the liver fragments are transferred to an extrahepatic location. In addition, it is impossible to use this model to study viral entry and neutralization since a real *in vivo* infection of Trimera mice transplanted with healthy human hepatocytes has never succeeded so far.

### 3.4 Chimeric uPA-SCID mice

#### 3.4.1. Characterization

Perhaps the most relevant small animal model for the study of hepatitis C is the chimeric uPA-SCID mouse. The urokinase-type plasminogen (uPA)-transgenic mouse was originally developed in 1990 by the group of Dr. Ralph Brinster for the study of plasminogen hyperactivation and the treatment of bleeding disorders [69]. Besides the high plasma uPA levels and hypofibrinogenemia, the liver-specific overexpression of the uPA-transgene also causes extensive hepatic toxicity and liver disease [70]. Due to the chronic liver disease and hepatic insufficiency, an environment is created that supports repopulation by healthy murine hepatocytes [71]. To prevent graft rejection after xenogeneic hepatocyte transplantation, the Alb-uPA mouse was backcrossed onto an immunodeficient mouse strain. This allowed for the repopulation of rat hepatocytes in Swiss athymic (nu/nu) Alb-uPA mice [72], and transplantation of uPA-RAG2 mice with hepatocytes of woodchuck origin [73,74].

The logical next step was to investigate whether immune deficient Alb-uPA mice could also be transplanted with human hepatocytes. Dandri *et al.* showed that this was indeed possible [75]. The amount of repopulation was however considerably lower than what was feasible with mouse or rat hepatocytes. While both these latter hepatocytes can nearly completely repopulate the diseased mouse liver, only about 15% of mouse liver parenchyma could become repopulated with human hepatocytes. There are different possible explanations for that. The type and duration of prior anticancer therapy of the donor and the period of ischemia during surgical removal of the liver fragment have a major influence on the quality of isolated human hepatocytes. In addition, it is not unreasonable to assume that the communication between human donor cells and the mouse environment occurs less optimal than with transplanted cells isolated from other rodent species. Another factor that has a major influence on the transplantation efficiency is the zygosity of the Alb-uPA mice. Heterozygous animals rapidly experience spontaneous somatic deletion of the transgene, thereby generating healthy mouse hepatocytes that can quickly repopulate the diseased liver and can be recognized as ‘red nodules’ [70]. The mouse hepatocytes have a growth advantage over the transplanted human hepatocytes and therefore stable and pronounced engraftment is prevented. While animals harboring relatively low levels of human hepatocytes in their liver can support a hepatitis B virus infection [75], they are not susceptible to HCV infection [76].

**Figure 1.**
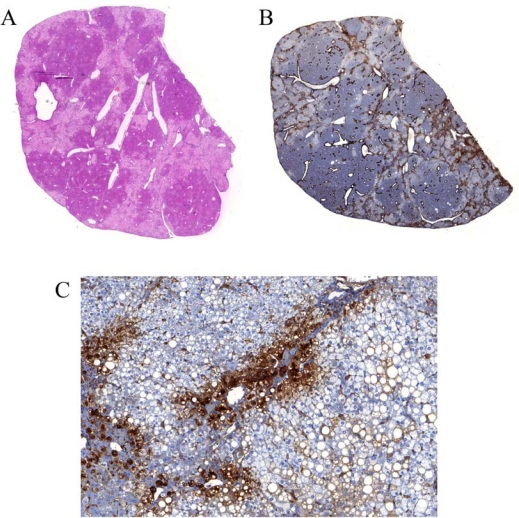
Immunohistochemical analysis of the liver of a uPA-SCID mouse transplanted with primary human hepatocytes. Pale regions within the liver parenchyma represent human hepatocytes, while the darker areas are occupied by mouse hepatocytes. Overview of a complete chimeric liver section stained with H&E (A) or an antibody against glutamine synthetase (B). (C) Magnification of a human region within (B) showing the preferential expression of glutamine synthetase near the centrolobular region of the liver lobule.

To study HCV infection, highly repopulated chimeric mice are needed. This can be achieved by transplanting animals that are homozygous for the uPA-transgene [76]. A fairly simple screening method can be used to shift the breeding colony for the production of uPA^+/+^-SCID mice [77]. These animals do not suffer from the spontaneous transgene deletion and are therefore much better recipients for xenotransplantation. Indeed, several weeks after transplantation with freshly isolated primary human hepatocytes, a high proportion of the diseased liver tissue, sometimes exceeding 90%, is replaced by human hepatocytes [78,79]. Interestingly, the repopulation of the diseased liver is a well-organized process. Only the human cells that make contact with diseased mouse tissue are actively proliferating and human canalicular structures are making functional connections with the mouse biliary system [79]. Within the human areas, the seemingly uniform hepatocytes acquire specialized functions depending on their localization within the liver lobule, a process called hepatic zonation [80]. Similar to what is observed in the healthy human liver, the enzyme glutamine synthetase (GS) is specifically expressed in the hepatocytes surrounding the centrolobular veins (Figure 1). This spatial expression pattern and functional heterogeneity are typical features of a complex organ like the liver.

Analysis of the plasma of chimeric mice clearly confirmed the synthetic functionality of the human hepatocytes. In addition to human albumin, which is used as a marker for successful repopulation, more than 20 other proteins of human hepatic origin, like α-1 antitrypsin, apolipoproteins, several clotting factors and complement proteins, could be identified [79,81]. The transplanted human hepatocytes also retain their natural detoxifying functions [78]. We have recently shown that the human metabolic profile of certain steroids is closely mimicked in chimeric mice [82,83]. This makes this animal model exceptionally useful for the study of metabolism and hepatotoxicity of newly developed medicinal compounds.

#### 3.4.2. HCV infection

The major advantage of highly repopulated mice is that these chimeric animals are now susceptible to HCV infection [76,79]. After inoculation with the virus, HCV RNA levels in the plasma rapidly increase and frequently reach levels exceeding 10^7^ IU/mL within two to three weeks. Plasma of these mice can be used to passage the infection to other chimeric mice. So far, we have already demonstrated the infectivity of eight consensus strains representing the six major genotypes (Bukh *et al.*, manuscript submitted). These consensus strains were all collected from acutely infected chimpanzees but we have also succeeded to infect chimeric mice with plasma from HCV infected patients. This is a major advantage over the currently available cell culture system, which is limited to one particular viral strain JFH1, or chimeric derivatives thereof [5,8].

Another major difference with the HCV cell culture system relates to the characteristics of the secreted virus. We have shown that the specific infectivity of virus produced by infected chimeric mice and chimpanzees is much higher than the infectivity of virus produced in cell culture [84]. This highly infectious virus has a lower average buoyant density. These differences may be related to the physical association of *in vivo* produced viral particles with low-density lipoproteins and suggests that at least the morphogenesis and secretion of viral particles in chimeric mice closely resembles that of a natural infection in chimpanzees and man. The authenticity of the characteristics of the viral particle is very important, especially in the context of the study of viral entry. It has been shown that lipoproteins can modulate the entry of (pseudo)viral particles *in vitro* [85–87].

HCV infected chimeric mice are not able to spontaneously clear the virus. This is not surprising since the uPA-SCID mouse lacks a functional adaptive immune system. Nevertheless, the innate immune system of the hepatocyte is directly activated after infection [88]. Microarray analyses showed that besides the activation of genes involved in the interferon response, HCV also manipulates genes implicated in other intracellular events like lipid metabolism and apoptosis induction [88,89]. Joyce *et al.* recently showed that HCV infected hepatocytes in chimeric mice suffer from oxidative and ER stress, which ultimately results in hepatic damage and increased apoptosis of the infected cells [89]. This was surprising since HCV, in contrast to HBV [90,91], is usually regarded as a non-cytopathic virus.

#### 3.4.3. Study of entry inhibitors

Since chimeric uPA-SCID mice lack a functional immune system they cannot be used to study directly the cellular and humoral immune response during infection or after vaccination. It is however possible to evaluate the efficacy of adaptive immune responses after passive transfer of e.g. antibodies. In chronic, but also in a fraction of acute HCV patients, antibodies with *in vitro* neutralizing activity have been identified [92,93]. Especially in chronic patients these antibodies seem to be capable to neutralize HCVpp and HCVcc of different genotypes. Since there is no clear relation between the presence of these neutralizing antibodies and the outcome of viral infection, the *in vivo* relevance of these antibodies has long been questioned. We have recently shown that passive immunization of chimeric mice with polyclonal antibodies from a chronic HCV patient (patient H) can indeed protect these mice from a subsequent challenge with the viral strain this patient was originally infected with [94]. A clear correlation was observed between the administered antibody dose and the fraction of animals that were protected. Chimeric mice that became infected experienced a delay in the kinetics of the viral load. Sequence analysis of the virus of infected animals showed that in one animal a variant had emerged with a mutation in the E1 protein. Subsequent *in vitro* assays showed that this mutant virus was still sensitive to neutralization. Recently, Law *et al.*, showed that chimeric mice could also be protected with monoclonal neutralizing antibodies [95]. Although huge amounts of antibodies needed to be administered before the treatment was efficacious, these experiments clearly showed the functionality of neutralizing antibodies and indicated their possible usefulness for the prevention of reinfection of chronic HCV patients after liver transplantation.

Perhaps a better strategy to prevent reinfection after liver transplantation is to target the viral receptor(s) rather than the virus. This approach may be more effective, given the high variability of the virus and the conserved nature of the cellular receptor. We have recently shown that antibodies that block CD81, one of the viral co-receptors, are effective in preventing HCV infection in chimeric mice [96]. A single dose of 400 μg of monoclonal anti-CD81 antibody, one day before and one day after virus injection, is sufficient to completely protect the challenged chimeric mice. As expected, this prophylactic treatment is effective against HCV of different genotypes. In a second experimental setup, the virus was injected six hours before the anti-CD81 therapy was initiated. This post-exposure therapy was unable to prevent or even delay the kinetics of the infection. Meanwhile, two groups independently demonstrated that HCV can spread *in vitro* directly from one infected hepatocyte to a neighboring cell via a CD81-independent route [97,98]. Nevertheless, an *ex vivo* saturation of the donor liver with CD81 antibodies or alternatively with specific chemical compounds may be a suitable strategy to protect the liver after transplantation in chronic HCV patients. This procedure could even be complemented with an antiviral therapy consisting of interferon, ribavirin and STAT-C. In addition, new studies should be undertaken to evaluate whether one of the other co-receptors such as SR-B1, Claudin-1 or Occludin are potential targets for antiviral therapy.

#### 3.4.4. Study of viral replication and translation

Besides the study of viral entry, chimeric mice have already proven useful to explore molecular aspects of viral replication and translation. In collaboration with Ralf Bartenschlager we studied the infection of chimeric mice with JFH1 variants that contain mutations in both the structural and nonstructural region of their genome [99]. These cell culture adapted variants yield higher titers of infectious particles and show an enhanced spread *in vitro*. Although these highly adapted viruses induce a robust infection in chimeric mice, sequence analysis of the progeny virus showed that the NS5A mutation, which was the major mutation responsible for the increased viral titer in the culture supernatant, had reverted to the wild type or an additional mutation arose in NS5A. This indicates that cell culture adaptive mutations result in an impaired fitness of the virus *in vivo*. This was not surprising since it was previously shown that Con1 genomes that carry replication-enhancing mutations (REMs) are severely attenuated in chimpanzees and also quickly revert to the wild type sequence [100]. In a recent study conducted with Ralf Bartenschlager, we demonstrated that the *in vivo* attenuation of REM-containing HCV genomes is caused by an interference at the level of virion assembly [101].

#### 3.4.5. Evaluation of antiviral compounds

While it is clear that newly developed antiviral compounds need to be tested in preclinical animal models before they can be evaluated in clinical trials in humans, the only suitable animal model for efficacy studies was the chimpanzee. Because of financial and ethical constraints limit efficacy studies in chimpanzees, pharmaceutical companies perform preclinical safety studies in a variety of animal species but limit the efficacy studies to experiments in cell culture systems. However, since all these cell culture assays rely on hepatoma cells and (sub)genomic replicons or the JHF1-strain, one cannot simply assume that the novel compounds will show the same antiviral effects in a genuine infection with ‘natural’ virus. Since chimeric mice uPA-SCID mice are repopulated with primary human hepatocytes and can be infected with natural HCV of different genotypes, they may bridge the gap between the *in vitro* studies and clinical trials in humans.

Several antiviral products have thus far been evaluated in this system [102]. Interestingly, interferon alpha therapy of genotype 3 infected mice was more effective than the same therapy in genotype 1 infected mice [103]. This correlates well with the more favorable therapeutic outcome of standard interferon therapy in genotype 3 infected patients. The chimeric mouse system has been further validated with several STAT-C compounds, like protease [104] and polymerase inhibitors [105].

Because this mouse model is based on the infection of primary human hepatocytes, one can also evaluate compounds that target cellular proteins. DEBIO-025, a non-immunosuppressive Cyclosporin A derivative, can inhibit the interaction between the cellular protein cyclophilin and the viral polymerase NS5B, thereby inhibiting viral replication *in vitro*. While DEBIO-025 monotherapy had no effect on the viral infection in chimeric mice, it had a synergistic effect when combined with pegylated interferon [106]. This cyclophilin inhibitor is currently under evaluation in clinical trials [107].

## 4. Conclusions and future perspectives

The chimpanzee has played a major role in our current understanding of the basic and clinical aspects of HCV infection, but ethical and financial constraints have understandably limited the use of this animal model. Of all the alternative species thus far investigated for their permissiveness to HCV, only the chimeric uPA-SCID mouse model seems to be a useful surrogate of, or at least a complement to, the chimpanzee. In fact, this chimeric small animal model has already contributed considerably to our knowledge of viral entry, replication and therapy. However, also this alternative animal model has its limitations. The surgical procedure to transplant human hepatocytes in two-week old mice is quite challenging, especially since the uPA-overexpression makes the animals extremely vulnerable to bleeding. In addition, the quality of the transplanted primary hepatocytes is of the utmost importance to generate highly repopulated animals, a prerequisite to achieve successful infection with HCV. All these issues result in a low production efficiency and correspondingly high cost.

The next challenge is to complement this immune deficient chimeric mouse with a functional human immune system. This would allow the study of human immune responses towards HCV and might result in a small animal model that may possibly replace the chimpanzee.
